# The Potential Role of iNOS in Ovarian Cancer Progression and Chemoresistance

**DOI:** 10.3390/ijms20071751

**Published:** 2019-04-09

**Authors:** Michal Kielbik, Izabela Szulc-Kielbik, Magdalena Klink

**Affiliations:** Institute of Medical Biology, Polish Academy of Sciences, 106 Lodowa Str., 93-232 Lodz, Poland; mkielbik@cbm.pan.pl (M.K.); iszulc@cbm.pan.pl (I.S.-K.)

**Keywords:** iNOS, ovarian cancer, chemoresistance

## Abstract

Inducible nitric oxide synthase (iNOS), the enzyme responsible for nitric oxide (NO) production, is not present in most cells under normal conditions. The expression of its mRNA, as well as its protein synthesis and full enzymatic activity, undergoes multilevel regulation including transcriptional and posttranscriptional mechanisms, the availability of iNOS substrate and cofactors and oxygen tension. However, in various malignant diseases, such as ovarian cancer, the intracellular mechanisms controlling iNOS are dysregulated, resulting in the permanent induction of iNOS expression and activation. The present review summarizes the multistaged processes occurring in normal cells that promote NO synthesis and focuses on factors regulating iNOS expression in ovarian cancer. The possible involvement of iNOS in the chemoresistance of ovarian cancer and its potential as a prognostic/predictive factor in the course of disease development are also reviewed. According to the available yet limited data, it is difficult to draw unequivocal conclusions on the pros and cons of iNOS in ovarian cancer. Most clinical data support the hypothesis that high levels of iNOS expression in ovarian tumors are associated with a greater risk of disease relapse and patient death. However, in vitro studies with various ovarian cancer cell lines indicate a correlation between a high level of iNOS expression and sensitivity to cisplatin.

## 1. Introduction

Inducible nitric oxide synthase (iNOS, NOS2) is one of three isoforms belonging to the family of nitric oxide synthases, which are enzymes that catalyze the production of nitric oxide (NO) from L-arginine. iNOS is a unique enzyme since the iNOS transcript and protein are not present under normal conditions in most cells. Its expression is inducible and is frequently associated with inflammation and malignant diseases. iNOS expression, enzyme activation, and subsequent NO production comprise a multistage process that undergoes complex regulation on many levels from mRNA induction to the modulation of full enzymatic activity. In the tumor environment, a number of stimuli are constantly present, and large molecular changes occur in tumor and stromal cells, resulting in the permanent induction of iNOS expression. It is well established that iNOS, along with derived NO, is an important factor in both protumor and antitumor activity, which has been proved by a number of papers available on this topic. While the importance of iNOS expression in ovarian tumors is not obvious and far from being fully understood, the present review summarizes its possible involvement in the development and growth of ovarian cancer, its association with the chemoresistance of ovarian cancer cells to platinum compounds, and its potential as a prognostic factor in the course of this disease.

## 2. iNOS—Structure, Enzymatic Activity and Regulation in Normal Cells

**Figure 1:**
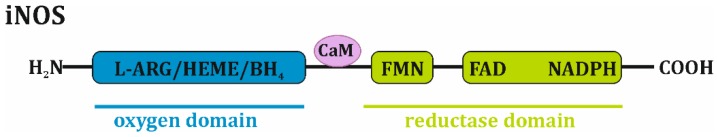
Domain-structured scheme of iNOS monomer. iNOS monomer is composed of oxygen domain that associates L-arginine (L-ARG), protoporphyrin IX (HEME) and tetrahydropterin (BH4), as well as reductase domain that consists of flavin mononucleotide (FMN), flavin adenine dinucleotide (FAD) and reduced form of nicotinamide adenine dinucleotide phosphate (NADPH). Calmodulin (CaM) is noncovalently bound to the iNOS complex.

Inducible nitric oxide synthase, which is similar to other NOS isoforms, is a homodimer with a molecular weight of approximately 130–135 kDa. It adopts a bidomain structure in which a carboxy-terminal “reductase” moiety is associated with flavin mononucleotide (FMN), flavin adenine dinucleotide (FAD) and the reduced form of nicotinamide adenine dinucleotide phosphate (NADPH), while an amino-terminal “oxygen” domain is a binding site for protoporphyrin IX (heme), tetrahydropterin (BH_4_) and L-arginine_._ Calmodulin is noncovalently bound to the iNOS complex [1,2] (Figure 1).

Monomeric iNOS is unable to bind the BH_4_ cofactor and the L-arginine substrate, thus, it cannot synthesize nitric oxide. The homodimerization process, which is required for enzyme activation, occurs in two oxygen domains, resulting in rapid conformational changes, and is dependent on the availability of L-arginine and BH_4._ Moreover, the presence of heme appears to be mandatory for dimer formation and to stabilize the whole enzymatic complex. Therefore, the physiological regulation of the enzymatic activity of iNOS is primarily based on the dietary intake of L-arginine, the regulation of its transport into cells, its use by other competing biological systems, such as arginase, and the regulation of BH_4_ synthesis and consumption. Moreover, iNOS catalytic activity is also downregulated by NO feedback since this molecule strongly binds to the heme group. The extracellular/pharmacological blocking of iNOS activity may occur in various manners and is mainly based on the use of L-arginine derivatives that compete with the substrate, the inhibition of NADPH, the inhibition of BH_4_ synthesis or binding, and the inhibition of heme binding [1,2,3,4,5,6].

It is well established that NO synthesis by iNOS includes two catalytic steps. First, L-arginine is hydroxylated by molecular oxygen (O_2_) and NADPH to N^ω^-hydroxy-L-arginine, which is next oxidized to L-citrulline, H_2_O and NO. Moreover, in the absence of L-arginine and BH_4_, the monomeric form of iNOS reduces molecular oxygen to the superoxide anion (·O_2_^−^). Thus, BH_4_ prevents superoxide anion synthesis via NOS. However, the reduction of oxygen to superoxide is an obligatory step during NO synthesis. The presence of both molecules (NO and ·O_2_^−^) in the reaction milieu leads to their rapid reaction to form the highly reactive radical peroxynitrite anion (ONOO^−^), which is characterized by strong nitrosative and oxidative properties. Another factor controlling iNOS enzymatic activity is oxygen tension since L-arginine is converted in an O_2_-dependent manner [1,4,7,8].

The induction of iNOS expression is a complex mechanism that undergoes multilevel control and is primarily regulated by both transcriptional and posttranscriptional mechanisms that are highly cell- and species-dependent. The human *iNOS* (*NOS2*) gene is 43,764 bp in size and located on chromosome 17. It encodes two transcripts: NOS2_001 and NOS2_201. The first transcript is 4176 nt in length, encodes a protein of 1153 amino acids and is thought to be a major *iNOS* mRNA. The NOS2_201 transcript is 4089 nt in length and encodes a protein of 1114 amino acids that is a splice variant of NOS2_001, but its involvement in protein synthesis is poorly recognized [5]. Extracellular signals control transcription factor activity and, consequently, *iNOS* gene expression and subsequent mRNA translocation. These extracellular signals are primarily pro-inflammatory cytokines (interleukin 1β, IL-1β; interferon γ, IFN-γ; tumor necrosis factor α, TNF-α), bacterial products (lipopolysaccharide, LPS; lipoarabinomannan, LAM), hypoxia and oxidative stress. The promoter sequence for human *iNOS* contains a TATA box approximately 30 bp from the transcription start site. Next to this are binding sites for transcription factors (NF-kB, NF-IL6), octamer factors, transcription factors induced by TNF-α, and transcription factors induced by IFN-γ (IRF-1, STAT-1α). It is worth noting that the maximal induction of the human *iNOS* gene is believed to occur through the binding of NF-κB and IRF-1 together to promoter sequences. Interestingly, a 1000 bp fragment of the human *iNOS* promoter showed low basal activity but no induction by cytokines. Only an *iNOS* promoter fragment larger than 3.8 kb showed any significant response to cytokines. Therefore, the promoter region relevant to cytokine-induced NF-κB/STAT-1α transcriptional factors for the human *iNOS* gene is located 3.8 kb upstream of the 5′ flanking region. A classical cytokine-induced enhancer is located between position 5.2 and 6.5. However, the notably high induction of iNOS expression (8-10-fold) was also found with the promoter fragment ranging from 7.2 kb to 16 kb [5,9,10,11]. Pathways that greatly contribute to transcriptional factor activation are the NF-κB signaling pathway, the Janus tyrosine kinase-Signal transducer and activator of transcription (JAK-STAT) pathway, and the mitogen-activated protein kinase (MAPK) pathway. Among them, NF-κB is a central factor for the activation or inhibition of *iNOS* gene expression. IL-1β, TNF-α, and oxidative stress induce NF-κB activity, its translocation to the nucleus and its subsequent binding to the promoter sequence. In contrast, transforming growth factor-β1 (TGF-β1) and antioxidants inhibit NF-κB activity through the induction of its ubiquitin-dependent proteasomal degradation, blocking its translocation to the nucleus or enhancing I-κB expression [5,11,12,13]. Interestingly, different polymorphisms in the sequence of the human *iNOS* promoter and their correlation with various human diseases, such as asthma or Alzheimer’s disease have been described, and the relationship between specific polymorphisms and iNOS expression, activity, and NO production [12] have been reported.

The posttranscriptional regulation of human *iNOS* mRNA primarily involves mRNA stability and/or degradation. The 3′-untranslated region (3′-UTR) of human *iNOS* contains four AUUUA motifs and one AUUUUA motif, which are known to destabilize the mRNAs of proto-oncogenes, nuclear transcription factors, and cytokines. AU-rich elements (ARE) mediate mRNA decay primarily by the protein requirement of exosomes containing mRNA. Moreover, the TGF-β-mediated activation of nucleases acting on AU-rich sequences leads to mRNA destabilization and the inhibition of iNOS induction. Therefore, the posttranscriptional regulation of mRNA stability is dependent on the 3′-UTR region. Moreover, several RNA-binding proteins (HuR, AUF1, KSPR, TIAR, TTP, PTB) are involved in either the stability or the degradation of human *iNOS* mRNA. Using DLD-1 cells, the only human model available for this type of research, it was shown that in unstimulated cells, KSPR binds to the 3′-UTR of *iNOS* mRNA and recruits exosomes to the mRNA, resulting in its rapid degradation. In cells stimulated with cytokines, TTP interacts with KSPR, preventing the binding of KSPR to mRNA, which in turn enhances HuR binding to the 3′-UTR *iNOS* sequence. The interaction of HuR with the 3′-UTR region increases *iNOS* mRNA stability and thus enhances iNOS expression. AUF1 consists of four isoforms, all of which can bind to ARE and promote *iNOS* mRNA degradation by competing with HuR for the same AU-rich elements. In contrast, TIAR enhances mRNA stability and increases iNOS expression [5,11,14,15,16].

Apart from effects on mRNA stability, another potential mechanism of iNOS posttranscriptional regulation involves short, noncoding RNAs known as microRNAs (miRNAs). It has been suggested that miRNAs block translation by the posttranscriptional repression of human iNOS. This was described in a study by Guo et al. [17], in which miR-939 was found to bind to the 3′-UTR in in vitro and in vivo models. miR-939 binding decreased cytokine-induced iNOS protein expression but did not affect its mRNA level and stability. Similarly, miR-26a and miR-146a affected the expression of iNOS protein either directly by interacting with the 3′-UTR (miR-26a) or indirectly by modulating the level of inflammatory cytokines (miR-146a) [18,19].

Finally, the posttranscriptional regulation of iNOS expression may involve mRNA translation and protein stability. Human cardiomyocytes express factors that can inhibit *iNOS* mRNA translation by interacting with the 5′- and/or 3′-UTR sequences of *iNOS* mRNA [20]. Furthermore, Felley-Bosco et al. [21] reported that the overexpression of caveolin-1 (Cav-1) in human DLD-1 cells destabilized iNOS. These data suggest that the direct interaction of Cav-1 with iNOS increased the proteasomal degradation of this enzyme.

Human iNOS expression and activity are observed in a large number of normal cells and various tissues, e.g., macrophages, neutrophils, Kupffer cells, chondrocytes, hepatocytes, and the vasculature [22]. Despite the complex, multilevel regulation of iNOS induction and activity, it is constantly upregulated in cancer cells, most likely due to vast alterations in their cellular biology.

## 3. iNOS Expression and Regulation in Ovarian Tumors

The constitutive overexpression of iNOS has been demonstrated in a number of tumors including breast, brain, prostate, lung, pancreas, ovarian, bladder, gastric, and colorectal tumors as well as melanoma and Kaposi’s sarcoma. In most tumors, it is possible to observe the increased expression and enzymatic activity of iNOS in comparison to those in adjacent normal tissue. However, it must be noted that the cellular level and activity of iNOS strongly depend on the histological type and grade of the tumor as well as the clinical stage of the disease. Moreover, the expression of iNOS in the tumor microenvironment (tumor cells along with stromal cells) varies primarily depending on the complexity of the particular tumor environment as well as on the presence of primary or metastatic lesions. Although its precise role has still not been fully established, iNOS-derived NO has a biphasic effect on tumor-related processes, such as tumorigenesis/malignant transformation, tumor progression, angiogenesis, metastasis, and chemoresistance. This topic has been widely discussed, with vast and comprehensive knowledge available in the form of reports and reviews, therefore it was not the purpose of this paper. The most important factors that strongly influence the dichotomous effects of NO are its concentration, duration, cell cycle status, and cell redox condition and the presence of oxygen and oxygen radicals that allow the formation of reactive nitrogen species (RNS) [23,24,25].

iNOS expression is significantly increased in ovarian cancer compared to its expression in normal ovarian tissue or benign tumors [26]. As determined in three groups of patients suffering from ovarian cystic tumors, 88% of malignant tumors were characterized by the strongly increased expression of iNOS in comparison to that in nonneoplastic (5%) or benign tumors (6%) [27]. The positive expression of iNOS was observed in both epithelial cells and tumor-associated macrophages (TAM) in malignant, borderline and benign tumors [28]. The importance of iNOS-positive stromal cells (omental adipose stromal cells) in the promotion of ovarian cancer cell proliferation and their resistance to paclitaxel was shown by Salimian Rizi et al. [29]. However, it should be emphasized that similar to other tumors, both iNOS and NO play rather multifaceted roles in ovarian cancer, and despite obtainable reports in this subject, it is difficult to draw any final conclusions [30].

**Figure 2:**
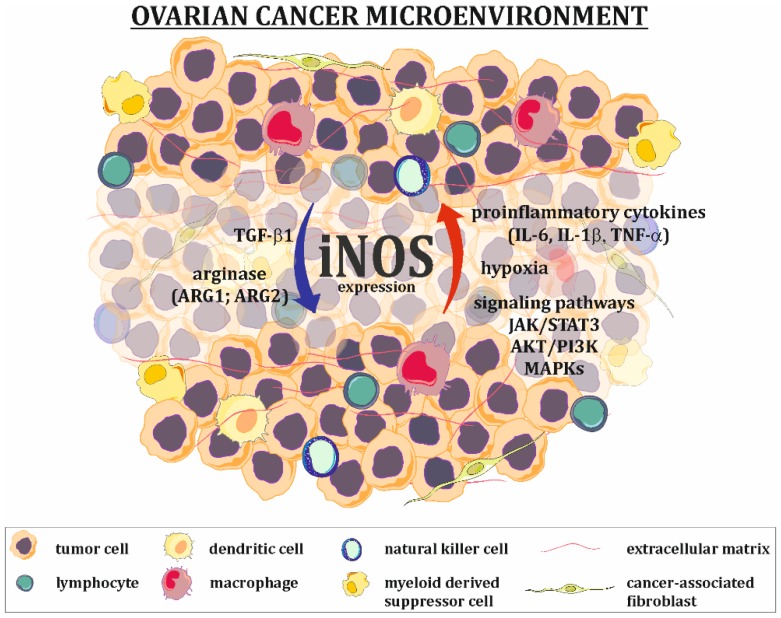
Regulation of iNOS expression in ovarian cancer microenvironment. Factors present in the ovarian cancer microenvironment, such as proinflammatory cytokines (IL-1β, IL-6, TNF-α), as well as hypoxia and overactivation of some signaling pathways (e.g., JAK/STAT3, AKT/PI3K, MAPKs), are responsible for upregulation of iNOS expression in tumor cells, while agents like TGF-β1 and arginase downregulate its expression.

The expression of iNOS in tumor cells is upregulated (Figure 2) by extracellular signals, such as pro-inflammatory cytokines and hypoxia. The ovarian cancer tumor microenvironment is characterized by high levels of IL-6, IL-1, and TNF-α, which strongly activate the JAK/STAT and MAPK signaling pathways known to be iNOS inducers [31,32]. It should be mentioned that cancer cells are characterized by the constant overexpression and overactivation of STAT3 [33], serine-threonine protein kinase (AKT) [34] and MAPK [35,36] signaling proteins, allowing them to survive, grow, proliferate and resist various chemotherapeutics. Many in vitro studies have shown that hypoxia alone or in parallel with pro-inflammatory cytokines increases the level of *iNOS* mRNA. The main mechanism for hypoxic iNOS stimulation includes two pathways. In the first pathway, hypoxia-inducible factor (HIF), an essential factor in the cellular response to hypoxic conditions, directly binds to the hypoxia-responsive element (HRE) present in the promoter region of *iNOS*. In the second pathway, hypoxia activates NF-κB through the activation of the inhibitory κB kinase, leading to classical NF-κB signal transduction and the induction of iNOS. It is worth noting that both of these pathways are interdependent and can be altered by multiple factors (including NO) [37].

The agents that downregulate iNOS expression/activation are primarily arginase and TGF-β1 (Figure 2). Arginase exists in two forms: ARG1 and ARG2. It catalyzes the conversion of arginine to ornithine and urea. One of the functions of arginase is the regulation of iNOS activity by lowering L-arginine bioavailability and the downregulation of iNOS expression, which is manifested by low levels of this amino acid [38,39]. The positive expression of TGF-β1 in ovarian tumors, as well as in ovarian cancer cells present in ascites fluid, has been well documented [40,41,42]. This cytokine suppresses the expression of iNOS at the mRNA level [43].

## 4. Implication of NO/RNS in the Development of Ovarian Cancer

**Figure 3:**
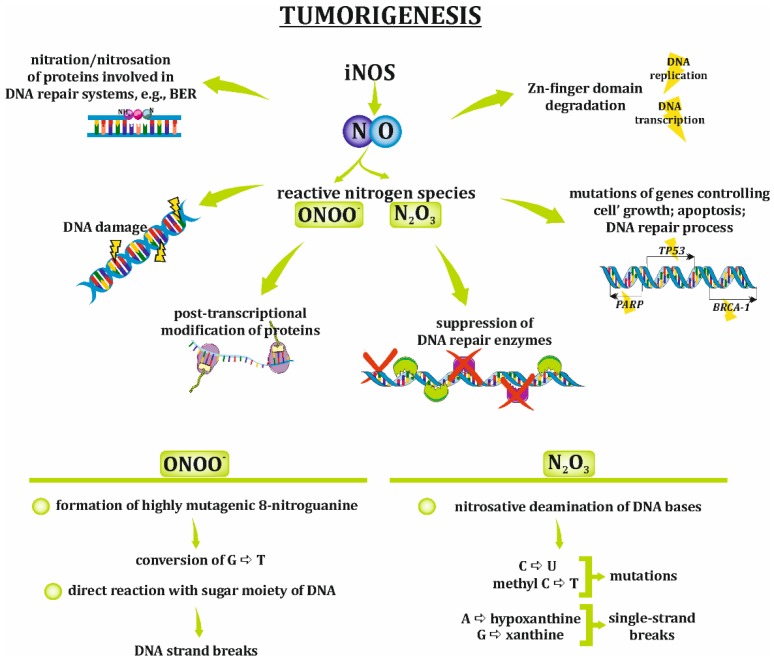
Role of NO and reactive nitrogen species in tumorigenesis. Although the role of iNOS has never been directly determined in carcinogenesis, the activity of its product—nitric oxide (NO)—and NO’s derivatives, such as peroxynitrite (ONOO^−^) or dinitrogen trioxide (N_2_O_3_), influence normal cells causing their transformation and inducing tumorigenesis. BER, base extinction repair system; G, guanine; A, adenosine; C, cytosine; U, uracil; T, thymidine.

Tumorigenesis/carcinogenesis requires multilevel modification in somatic cells, resulting in the alternation of their morphology and functional features, such as their unlimited proliferative potential or insensitivity for apoptotic signals. The role of nitric oxide synthases, particularly iNOS, in carcinogenesis has never been directly determined, but the impact of NO, the product of these synthases, has been described in many reports. The multifaceted activities of NO or its derivatives, such as peroxynitrite and dinitrogen trioxide (N_2_O_3_), bring about different changes in normal cells, resulting in their transformation (Figure 3). The mechanisms by which NO and/or RNS induce tumorigenesis are strongly related to oxidative/nitrosative stress and include DNA damage, the suppression of DNA repair enzymes, posttranslational modification of proteins and the formation of nitrosamines [24,25]. Genetic disorders are a high-risk factor for the development of ovarian cancer, and various signals that induce DNA injury or interference with DNA repair processes are important causes of ovarian tumorigenesis [44]. Mutations in several genes, namely, *BRCA1* and *BRCA2* [45], mismatch repair genes [46], *BRAF, KRAS* [47], and *TP53* [48] are strong genetic risk factors for ovarian cancer promotion. Moreover, some authors have noted that *BRCA1* and *BRCA2* mutations are responsible for the development of almost 90% of all ovarian cancer cases [44].

The effect of NO and RNS on DNA leading to its damage, e.g., the induction of mutations, is rather well recognized and is summarized below. The exposure of DNA to NO/RNS results in the oxidation and nitration/nitrosation of the nucleic acid bases. For instance, peroxynitrite can form DNA-damaging 8-nitroguanine and 8-oxo-7,8-dihydro-2′-deoxyguanosine, which are biomarkers of inflammation-induced carcinogenesis. 8-nitroguanine is a highly mutagenic molecule able to convert guanine to tyrosine. Moreover, it was well described that ONOO^−^ induce a DNA strand breaks due to sugar fragmentation. Whereas, the dinitrogen trioxide causes nitrosative deamination of cytosine and methyl cytosine, which results in mutations, as well as the formation of hypoxanthine and xanthine. These compounds are very unstable and cause rapid depurination and single-strand breaks. Moreover, NO/RNS degrade zinc finger domains and, thus, strongly interfere with DNA replication and transcription. Genes controlling cell growth, DNA repair processes or apoptosis (e.g., *TP53*, *BRCA1*, *BRCA2*, *PARP*) are prime targets for NO/RNS. A strong relationship between iNOS activity and the G:C to A:T mutation in *TP53* resulting in the loss of p53 suppressor activity was reported in various carcinomas, e.g., colon, gastric, head and neck, and ovarian cancers [22,24,48,49,50,51].

Furthermore, RNS strongly participate in the nitration/nitrosation of DNA repair proteins. The formation of nitro adducts on aromatic groups, such as tyrosyl residues, and RNS attacks on nucleophilic sites, such as cysteinyl thiolates, are critical for protein function. The best-known targets for RNS are proteins involved in the base excision repair (BER) pathway. The inhibition of DNA repair protein activity makes cells more vulnerable to various carcinogenic mediators, e.g., alkylating agents and RNS [24,52,53].

However, it must be emphasized that the origin and causes of ovarian tumors still remain under debate. Thus, the NO/ effect of NO/RNS on DNA should be considered only as one of many possible mechanisms.

## 5. Can iNOS Expression be a Prognostic Factor in Ovarian Cancer?

Prognostic markers, which are well established in clinical practice, are mainly clinicopathologic in nature and include evaluation of the histopathological type, the stage of disease, the presence of high-volume ascites, the age at diagnosis and the process of surgical debulking [54,55,56]. Additionally, characteristic features of cancer cells, such as *BRCA* gene mutation [57], the surface molecule expression of EpCAM [58], and the protein expression of PTEN, HIF, and VEGF [59], are also useful as prognostic markers. Nevertheless, their effectiveness and accuracy are still not satisfactory to stop searching for new, more appropriate factors. Available reports regarding iNOS expression as a potential prognostic factor in ovarian cancer patients are rather controversial, and it is difficult to draw a final conclusion. The published data are conflicting and show both potential and the lack of prognostic value of iNOS expression. The following paragraphs present a short review of selected reports (published over the last 15 years) describing whether iNOS expression can be considered an important marker (Table 1).

### 5.1. Votes For

Raspollini et al. [60] studied the expression of iNOS (immunohistochemistry assay) in specimens obtained by surgical resection from 78 patients with stage III (FIGO classification) ovarian serous carcinoma with a low grade of differentiation (G3). In 50 patients (60%), positive immunostaining for iNOS was observed. Moreover, the authors proved that iNOS expression was significantly associated with the risk of disease relapse and patient death. In iNOS-positive patients, a disease-free survival period lower than 12 months accounted for 80% of patient outcomes, while in iNOS-negative patients, it accounted for 42%. The authors concluded that iNOS expression in ovarian tumors can be a significant independent predictor of disease relapse and patient survival.

The results of other studies [61] also clearly indicated the involvement of iNOS in poor patient survival. A total of 213 ovarian cancer patients enrolled in the studies were divided into two groups according to histopathology: type I (82 cases) and type II (131 cases). iNOS expression (immunohistochemistry) was observed in both groups, and there was significantly higher iNOS expression in patients with type II ovarian cancer compared to that in patients with type I ovarian cancer. Moreover, women with type II ovarian cancer had a poorer median survival time (60 months) than those with type I tumors (141 months).

Engels et al. [62] reported that iNOS is a prognostic marker for the clinical outcome of serous ovarian cancer patients. This study included a homogenous group of 112 patients with serous adenocarcinoma of the ovary. iNOS-negative tumors were characterized by improved progression-free survival time. Hence, pretherapeutic assessment of the iNOS level as a predictive factor for complete tumor resection might be of clinical value.

Other studies [63] tested the expression of iNOS in specimens from 90 patients categorized as benign, borderline, and malignant tumors by immunohistochemistry. As described, the highest expression of iNOS was observed in serous and mucinous malignant tumors, which was significantly higher than its expression in serous and mucinous benign tumors. The authors hypothesized that the upregulation of iNOS indicated its involvement in disease progression. Moreover, the enhanced expression of iNOS in less differentiated cancers may indicate its association with poor prognosis for patients with this type of tumor.

A positive correlation between high iNOS expression with poorly differentiated, advanced clinical stage (FIGO III/IV) serous ovarian carcinoma was shown by Li et al. [64]. The data were obtained based on an analysis of specimens from 150 ovarian tumor patients. iNOS overexpression plays an important role in the regulation of glycolysis, and the data show that iNOS-induced NO promotes this process. As the authors concluded, high levels of iNOS expression promote more aggressive phenotypes of ovarian tumors and are associated with poor survival outcome.

### 5.2. Votes Against

Very interesting and widely ranging studies were completed by Anttila et al. [65]. iNOS expression was tested in specimens from 301 epithelial ovarian cancer patients with consideration to FIGO stage, histopathological type, and histological grade. iNOS expression was correlated with histological subtype, and high levels of iNOS expression were only found in mucinous tumors. The FIGO and grade classifications, however, were not significant. Moreover, iNOS expression and its intensity had no prognostic value in any group of patients.

In studies published by Ozel et al. [66] iNOS expression along with angiogenesis microvessel density (MVD) were evaluated in specimens from 100 patients suffering ovarian carcinomas (various histopathological types). No correlation was found between iNOS expression and MVD, and iNOS expression had no impact on the survival of the patients.

Martins Filho et al. [67] evaluated 40 patients with ovarian cancer who underwent surgical treatment. Immunohistochemistry for iNOS and various cytokines was performed. The authors presented the complex relationship between iNOS, the immune response and tumor progression, indicating that there is no relationship between iNOS expression and the clinical stage of disease (FIGO), histological grade, response to chemotherapy, and disease-free survival ≤ 24 months.

### 5.3. NO Correct Answer

The examples presented above clearly show that the role of iNOS in ovarian cancer is not straightforward. Moreover, it is difficult to clearly see the whole picture, since all available information on this topic is rather modest due to the limited number of papers. Additionally, the lack of a sufficiently large group of patient samples, as well as the high histopathological variability of ovarian cancer, are another huge limitation in drawing unambiguous conclusions.

## 6. iNOS Expression Versus Chemoresistance in Ovarian Cancer Cells

The conventional therapy for ovarian cancer involves cytoreductive surgery of the tumor followed by the administration of chemotherapeutics. The accepted standard first-line treatment includes a combination of a platinum-containing agent (cisplatin, carboplatin) and a taxane (paclitaxel, docetaxel). The initial response to chemotherapy is promising, with the majority of patients (70–80%) responding positively. Nevertheless, depending on the stage of the disease, ovarian cancer recurs, with recurrence in up to 30% of patients with early-stage ovarian cancer to up to 85% of patients with an advanced stage of disease for 6–24 months after chemotherapy. Most relapse patients have acquired platinum resistance during repeated chemotherapy cycles. Moreover, in the heterogenic tumor microenvironment, some primary platinum-resistant cells are also present. It is accepted that prognosis for the five-year survival rate for patients with an advanced stage of the disease is rather poor and reaches approximately only 20%–35% [68,69].

**Figure 4:**
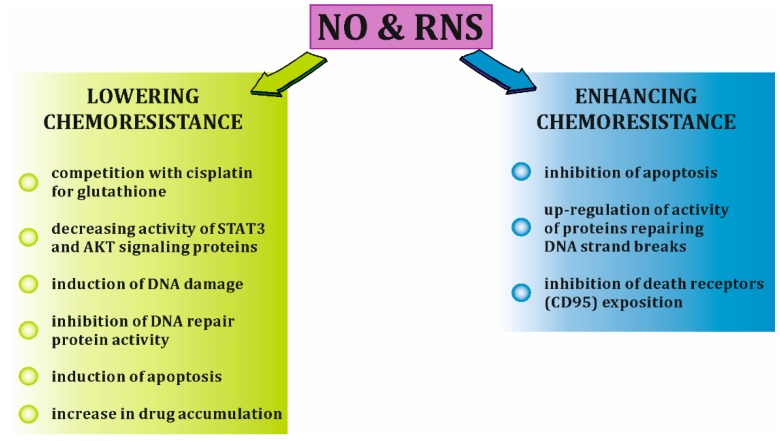
Selected mechanisms of NO/RNS impact on cisplatin resistance in cancer cells. RNS, reactive nitrogen species; STAT3, signal transducer and activator or transcription pathways 3; AKT, serine-threonine protein kinase.

The development of platinum resistance in ovarian cancer cells is a complex process. The molecular mechanisms implicated in this phenomenon include increased DNA repair, increased levels and activity of efflux pump proteins, the overactivation of signaling pathways (JAK/STAT, PI3K/AKT, MAPK) involved in cell survival, and cisplatin inactivation through the intracellular thiol glutathione [69,70]. iNOS-derived NO and its RNS derivatives can be implicated in all these processes in both ways, either enhancing or decreasing the platinum resistance. Lowering ovarian cancer chemoresistance by NO/RNS involves, among others competition with cisplatin for the glutathione [71], decreasing activity of STAT3 and AKT signaling proteins [48,72], induction of DNA damage [50,51,52], inhibition of DNA repair protein activity [24,52,53], induction of apoptosis through, e.g., activation of p53 pathway, down-regulation of anti-apoptotic proteins or activation of Fas receptor [25,73]. On the other hand, NO/RNS can enhance drug resistance of cancer cells by various mechanisms, such as inhibition of apoptosis through, e.g., inhibition of caspase activity or increase in Bcl-2 expression [22], up-regulation of activity of proteins repairing DNA strand breaks [74] or lowering death receptors (CD95) exposure on cell surface by cGMP-dependent phosphorylation of syntaxin 4 [75] [Figure 4].

Since chemoresistance in ovarian cancer is a serious clinical problem that has not been successfully overcome, there is an urgent need for a better understanding of its molecular basis. Thus, iNOS expression and activity and how they correlate with resistance to cisplatin have been the focus of many studies. A brief insight into the data available on this issue is presented below (Table 2).

In vitro studies on various ovarian cancer cell lines have delivered controversial data. For example, the cytosolic level of iNOS was significantly higher in the cisplatin-sensitive ovarian cancer cell line OV2008 than in resistant C13* cells, which suggests the association of cisplatin resistance with low iNOS content. Moreover, cisplatin significantly increased the iNOS level, but only in OV2008 cells. Interestingly, studies using a specific iNOS inhibitor clearly indicate that this protein participates in cisplatin-induced apoptosis in sensitive cells. The pro-apoptotic effect of iNOS is dependent on the accumulation of a high level of p53, which is caused by NO production [76]. A similar relationship between high basal iNOS expression and sensitivity to cisplatin was observed in SKOV-3 cells by Yu et al. [77]. This drug enhanced iNOS protein levels more efficiently in sensitive SKOV-3 cells in a dose-dependent manner than in resistant SKOV-3/DDP cells. Moreover, the increase in iNOS expression by using TAT-IDPs in SKOV-3/DDP cells resulted in increased apoptosis. In contrast, another study demonstrated that the cisplatin-resistant ovarian cancer cell line MDAH2774 was characterized by increased *iNOS* mRNA levels compared to the levels in its sensitive counterpart. However, yet another study showed that resistant and sensitive SKOV-3 cells manifested similar levels of *iNOS* mRNA [78]. iNOS-induced NO production was also observed in cisplatin-resistant ovarian cancer cells. Moreover, these cells are characterized by an enhanced level of glutathione, which is related to lower cisplatin efficacy, as high levels of this thiol are known to inactivate various cytotoxic agents [71]. Taken together, many reports support the argument that iNOS-expressing ovarian tumor cells are more sensitive to cisplatin chemotherapy and more easily undergo apoptosis than cells that do not express iNOS.

## 7. Targeting of iNOS in Ovarian Cancer

Since iNOS and the NO it produces have a dual nature and express both pro- and antitumor activity, it is probable that iNOS inhibitors or enhancers can be both beneficial and detrimental to ovarian cancer cell activity and survival. However, to date, few studies have explored the role of iNOS level manipulation in ovarian cancer. Thus, more research regarding the importance of this enzyme as a therapeutic target for ovarian cancer treatment is strongly needed. In a published report, the culture of SKOV-3 and MDAH2774 ovarian cancer cells with L-NAME (an L-arginine analog) significantly reduced the ability of cells to produce vascular endothelial growth factor (VEGF) and completely blocked their capacity for angiogenesis, in the in vitro angiogenesis assay. Therefore, the authors noted the positive effect of iNOS inhibition on blocking the metastatic potential of ovarian cancer cells. It should be noticed that the action of L-NAME was related to the decrease of the NO level, which is known to up-regulate the strongly pro-angiogenic factors IL-8 and VEGF-A [79]. Another study showed that a lack of iNOS activity is worse for cancer cells. The addition of 1400W, an iNOS inhibitor, to OVCAR-3 and Caov-3 ovarian cancer cell cultures or their treatment with *NOS2* siRNA significantly reduced cell growth. Moreover, targeting iNOS also had a positive effect on the immune cell content in the tumor environment, since *NOS2* siRNA decreased the number of M2-type TAM in OVCAR-3 and Caov-3 xenografts [80]. Similarly, silencing *iNOS* gene expression in epithelial the ovarian cancer cell lines MDAH2774 and SKOV-3 resulted in increased caspase-3 activity and a significant increase in cell apoptosis assessed by TUNEL. What is also interesting iNOS expression was in strict correlation with myeloperoxidase (MPO) expression, and silencing *MPO* gene also resulted in significant induction of ovarian cancer cells apoptosis [81]. On the other hand, it should also be mentioned that the beneficial effect of iNOS expression in tumor relapse was also described. Studies using a murine ovarian carcinoma model showed that IFN-γ gene therapy (liposomal IFN-γ gene) together with cisplatin-induced high levels of NO in ascites and kept the mice alive. In contrast, *iNOS* KO mice treated in the same way showed no NO in ascites and died in the course of treatment [82]. A different study also showed that iNOS-expressing micro-encapsulated cells significantly inhibited tumor mass (induced by SKOV-3) in mice [83]. Subsequent studies confirmed the efficacy of the induced activity of iNOS in tumor treatment. The tumor burden (mouse ovarian teratoma) was decreased, and the survival of mice was extended in the group of animals treated with IFN-β, which was accompanied by the increased level of NO. Moreover, the increased number of natural killer cells and macrophages in the tumor microenvironment of IFN-β-treated animals was observed [84]. In another study authors used two ovarian carcinoma cell lines, one parental ES2 cells, and one DLX4-overexpressing ES2 cells. They demonstrated that DLX4 (a homeoprotein) strongly stimulated the activity of STAT1 which next induced iNOS expression and high NO production. Whereas NO was responsible for the stimulation of ovarian cancer cells to high VEGF-A production. The authors also gave proof in the in vivo xenograft model that DLX4-related iNOS expression stimulated ovarian tumor angiogenesis. The key mechanism by which NO promote angiogenesis is the induction of VEGF-A production [85] (Table 3).

Although studies cited above clearly indicated that targeting iNOS therapy is promising in the treatment of ovarian cancer, available data on this topic are still scarce, and it is difficult to draw a final conclusion. To our best knowledge, there are no data about the clinical use of iNOS inhibitors/enhancers in ovarian cancer patients. The only available insight in this matter involves in vitro studies with cell lines and in vivo studies using animal models.

## 8. Concluding Remarks

iNOS expression is easily detected in ovarian tumor specimens and in various ovarian cancer cell lines cultured in vitro. Although the constant overexpression of this enzyme is often observed in this type of cancer, the role of iNOS in ovarian cancer growth, survival and resistance to platinum compounds is not clear. The multiple pros and cons of iNOS expression described in the available literature so far do not allow us to draw straightforward conclusions regarding the role of iNOS in ovarian tumors. While most reports point to the association of high levels of iNOS expression in ovarian tumors with the risk of disease relapse and patient death, some reports indicate that ovarian tumor cells with high iNOS expression are more sensitive to cisplatin treatment. The inhibition or induction of iNOS as a novel approach to ovarian cancer treatment also delivers conflicting results. Further studies on a large group of patients including a study on the histopathological variability of ovarian cancer and the determination of the molecular interplay between iNOS and various cancer cell-related signaling pathways are necessary to resolve the question of whether iNOS is beneficial or detrimental in ovarian cancer.

## Figures and Tables

**Table 1:** Possibility of using iNOS expression in ovarian tumors as prognostic/prediction factor.

**Votes For**	**Ref.**
iNOS expression in ovarian serous carcinoma (FIGO III) is an independent predictor of disease relapse and patients death	[60]
High iNOS expression in type II ovarian cancer is a poor prognostic factor	[61]
iNOS-negative ovarian serous carcinoma indicate longer progression-free survival	[62]
High iNOS expression in ovarian serous and mucinous carcinoma is a poor prognostic factor	[63]
High iNOS expression in ovarian serous and mucinous carcinoma is associated with more aggressive phenotype of tumor and poor survival outcome	[64]
**Votes Against**	
iNOS expression does not correlate with FIGO and grade and has no prognostic value	[65]
iNOS expression has no impact on patients survival	[66]
iNOS expression has no impact on FIGO, grade, response to chemotherapy or patients survival	[67]

**Table 2:** Correlation of iNOS expression and/or NO production with resistance of ovarian cancer cell lines to cisplatin.

Basal iNOS Expression, NO Production		Cisplatin-Induced iNOS Expression, NO Production		Ref.
Cisplatin Resistant Ovarian Cancer Cell Line	Cisplatin Sensitive Ovarian Cancer Cell Lines	Cisplatin Resistant Ovarian Cancer Cell Line	Cisplatin Sensitive Ovarian Cancer Cell Lines	
Low protein level	High protein level	No induction of iNOS	High induction of iNOS	[76]
Low protein level	High protein level	Low induction of iNOS	High induction of iNOS	[77]
High mRNA level	Low mRNA level	Nd	nd	[78]
Low mRNA level	Low mRNA level	Nd	nd	[78]
High NO production	Low NO production	Nd	nd	[71]

**Table 3:** Summary of the beneficial and detrimental effects of iNOS targeting in ovarian cancer.

Ovarian Cancer Model	Treatment	Effect	Ref.
**iNOS Inhibition**			
Ovarian cancer cell lines	L-NAME -NOS inhibitor	Reduction of VEGF production	[79]
Ovarian cancer cell lines	1400W- iNOS inhibitor *iNOS* siRNA	Inhibition of cell growth	[80]
Ovarian cancer cell lines	*iNOS* siRNA	Increased activity of caspase 3 Induction of cell apoptosis	[81]
Xenograft model	*iNOS* siRNA	Decrease in number of M2-type of TAM in tumor	[80]
**iNOS Induction**			
Murine ovarian carcinoma	IFN-γ + cisplatin	High level of NO in ascites Enhanced survival of mice	[82]
Murine ovarian carcinoma	iNOS-expressed micro-encapsulated cells	Inhibition of tumor growth	[83]
Mouse ovarian teratoma	IFN-β	Inhibition of tumor growth Enhanced survival of mice	[84]
Ovarian cancer cell lines	DLX4	Stimulation of STAT1 activity Stimulation of VEGF-A production	[85]
Xenograft model	DLX4	Stimulation of tumor angiogenesis	[85]

## Abbreviations

·O_2_^-^	superoxide anion
3′-UTR	3′-untranslated region
AKT	serine-threonine protein kinase
ARE	AU-rich element
BH_4_	tetrahydropterin
FAD	flavin adenine dinucleotide
FIGO	International Federation of Gynecology and Obstetrics
FMN	flavin mononucleotide
HIF	hypoxia-inducible factor
IFN-γ	interferon-γ
IL	interleukin
iNOS	inducible nitric oxide synthase
JAK	Janus tyrosine kinase
MAPK	mitogen-activated protein kinase
miRNA	microRNA
NADPH	reduced form of nicotinamide adenine dinucleotide phosphate
NO	nitric oxide
ONOO^-^	peroxynitrite anion
RNS	reactive nitrogen species
STAT	signal transducer and activator of transcription
TGF-β1	transforming growth factor-β1
TNF-α	tumor necrosis factor-α
